# PRO*BPD: effectiveness of outpatient treatment programs for borderline personality disorder: a comparison of Schema therapy and dialectical behavior therapy: study protocol for a randomized trial

**DOI:** 10.1186/s12888-018-1905-6

**Published:** 2018-10-19

**Authors:** Eva Fassbinder, Nele Assmann, Anja Schaich, Kristin Heinecke, Till Wagner, Valerija Sipos, Kamila Jauch-Chara, Michael Hüppe, Arnoud Arntz, Ulrich Schweiger

**Affiliations:** 1 Department of Psychiatry and Psychotherapy, University of Lübeck, Ratzeburger Allee 160, 23538 Lübeck, Germany; 2 Department of Anaesthesiology, University of Lübeck, Lübeck, Germany; 3 Department of Clinical Psychology, University of Amsterdam, Amsterdam, The Netherlands

**Keywords:** Borderline personality disorder, Psychotherapy, Dialectical behavior therapy, Schema therapy, Randomized controlled trial, Economic evaluation, Clinical effectiveness

## Abstract

**Background:**

Borderline Personality Disorder (BPD) is a severe mental illness that is associated with low quality of life, low psychosocial functioning, and high societal costs. Treatments for BPD have improved in the last decades. Dialectical behavior therapy (DBT) and Schema therapy (ST) have demonstrated efficacy in reducing BPD symptoms and costs. However, research has not compared these two treatment approaches. In addition, there is a lack of ‘real world studies’ that replicate positive findings in regular mental healthcare settings. Thus, the PROgrams for Borderline Personality Disorder (PRO*BPD) study will compare the (cost-) effectiveness of DBT and ST in structured outpatient treatment programs in the routine clinical setting of an outpatient clinic.

**Methods/Design:**

We aim to recruit 160 BPD patients, who will be randomly assigned to either DBT or ST. In both conditions, patients receive one group therapy and one individual therapy session/week for a maximum of 18 months. Both treatment programs have similar frameworks, which guarantee clinical equipoise. The primary outcome is a reduction of BPD-symptoms. Also, the costs related to BPD are assessed and an economic evaluation is performed from a societal perspective. Secondary outcomes examine other measures of BPD-typical and general psychopathology, comorbidity, quality of life, psychosocial functioning and participation. Data are collected prior to the beginning therapy and every six months until the end of therapy, as well as at six months, one year and two years of follow-up after the end of therapy. Finally, we conduct a qualitative study to understand patients’ experiences with the two methods.

**Discussion:**

The PRO*BPD study is the first randomized trial to compare the (cost-) effectiveness of DBT and ST. By examining the clinical effectiveness of a broad spectrum of outcome parameters, conducting an economic evaluation and assessing patients’ experiences, this study will significantly advance our knowledge on psychotherapy for BPD and will provide insight into the treatment approaches that should be offered to different BPD patients from clinical, economic and stakeholder’s perspectives.

**Trial registration:**

German Clinical Trial Register, DRKS00011534, Date of registration: 11/01/2017, retrospectively registered.

## Background

Borderline Personality Disorder (BPD) is a prevalent, severe, complex mental illness that is characterized by a pervasive pattern of instability in interpersonal relationships, self-image, affect, and behavioral dysregulation [1]. Patients with BPD suffer from an array of comorbidities with other mental disorders, e.g., depression, anxiety, substance use and other personality disorders [2]. BPD affects every aspect of life (e.g., relationships with others, education, work, self-care) and thus is associated with significant impairments in quality of life across mental, social and physical dimensions [3]; persistent poor social functioning and participation [4–8]; and high societal costs [9, 10].

The prevalence of BPD is estimated to be 1.1% in the general population [11]. BPD is one of the most frequent PD in clinical populations and is diagnosed in up to 10% of outpatient and 25% of inpatient populations [12–14]. Individuals who have BPD often experience crises with self-harm or suicide attempts, and thus show an intensive use of health services and high costs [11, 15–17]. The direct health care costs that result from BPD are markedly higher than those for depression (e.g., 11,817 € vs. 6058 € per patient two years after an index diagnosis in a German study) [17]. A recent systematic review and cost offset analysis from economic evaluations revealed that empirically supported psychological treatments for BPD can massively reduce those costs [16].

Longitudinal research has yielded important findings on the course of BPD: the symptoms that are related to impulsivity, such as self-harm and suicidality, are more quickly resolved. By contrast, affective symptoms that reflect chronic dysphoria, such as loneliness and emptiness, are more stable [18]. On the one hand, high rates of remission have been found after several years, in which patients no longer meet the full spectrum of criteria for BPD. However, severe impairments in social functioning and quality of life usually persist [5, 8].

Not long ago, BPD was viewed as an ‘untreatable’ disorder; however, it can now be successfully treated with several empirically supported psychotherapeutic methods from different therapeutic orientations that were specifically designed for BPD [19–21]. These treatments include Dialectical Behavior Therapy (DBT) [22, 23] and Schema therapy (ST) [24, 25], which are grounded in cognitive behavior therapy.

To date, DBT is the most frequently evaluated psychotherapy for BPD: A recent meta-analysis, a Cochrane Review on general psychotherapy for BPD, and a meta-analysis on DBT only summarize the evidence of the efficacy of DBT in treating patients with BPD, which has been demonstrated in several randomized controlled trials (RCT) [20, 26, 27]. The primary outcomes are decreases in suicidality, self-injuring and impulsive behaviors, emergency room visits and inpatient admissions. Effectiveness studies under routine clinical care conditions in Germany and Australia had comparable results and suggest that the findings generalize to ‘real world’ conditions [28, 29]. The German study also found that the DBT program was associated with substantial cost savings [30]. DBT has also been effective in treating BPD with comorbidities and other psychiatric conditions, such as substance misuse [31–33], eating disorders [34–36], posttraumatic stress disorder [37–40], or depression [41].

ST shows promise for treating BPD as it was effective in reducing all BPD criteria and led to substantial improvements in quality of life [42, 43]. Two RCTs [44, 45], one case series [46], five open pilot studies [47–49] and one implementation study [50] demonstrated decreases in all nine BPD symptoms, general psychiatric symptoms, and quality of life, as well as low treatment drop out. In the first Dutch RCT ST was compared with transference focused therapy (TFP), both offered in an individual design. ST showed better treatment retention, and in the intention-to-treat-analysis it was clinically more effective than TFP [45]. Also, ST was more cost-effective [51]. A pre-post comparison demonstrated successful implementation of individual ST under routine clinical care conditions [50]. A group format (Group schema therapy, GST) was developed by Farrell and Shaw [52] and was successfully tested in an RCT in the United States: Patients who received GST showed no drop-out, high BPD remission rates, significant reductions in BPD-typical and general psychiatric symptoms as well as improvements in psychosocial functioning, with large effect sizes after only 8 months. Two outpatient pilot studies on GST in the Netherlands [47] and Germany [48] used GST in combination with individual ST and found substantial improvements in BPD-symptoms, general psychopathology, quality of life and happiness. To systematically investigate the clinical effectiveness and cost-effectiveness of GST for BPD and to test different formats of GST (GST only vs. a combination of GST with individual ST), a large, international, multicenter RCT on GST for BPD is currently in progress [53]. A meta-analysis from 2013 (including all published outpatient studies through 2013, which are all mentioned above, except for [48, 49]) revealed an overall effect size of d = 2.38 for pre-post change and an overall drop-out rate of 10% for ST in BPD patients. This is a very low drop-out rate compared to the average drop-out rate of 25% for BPD patients for interventions with a minimum duration of 12 months [54].

In sum, there are promising findings on the clinical efficacy and effectiveness of DBT and ST in treating BPD [20]. Moreover, both treatments lead to impressive reductions in direct and indirect health care costs (approx. 10,000 € per patient per year) [30, 51]. However, research has not yet compared these two methods. A treatment comparison would be interesting for psychotherapy research because both treatments have different approaches to treating BPD and focus on different techniques, despite several common factors (see also methods and a detailed overview in [55]).

The objective of this study is to compare the (cost)-effectiveness of DBT and ST in patients who have BPD. The PRO*BPD trial is the first randomized trial to compare these two methods. The primary hypothesis is that DBT and ST significantly differ in reducing BPD-severity (two-sided hypothesis). A two-sided hypothesis was chosen because information from literature does not allow predictions on the directionality of the difference (see discussion for more information on this theme).

In addition, we evaluate the costs and perform an economic evaluation from a societal perspective. Thus, a secondary hypothesis is that the two methods differ in their cost-effectiveness. In a budget-constrained health care system, there is an urgent need for detailed information on the costs and benefits of an intervention to inform decisions on resource allocation. A major challenge for BPD-specific psychotherapy is to demonstrate that the treatment leads above and beyond reductions in external BPD-symptoms (e.g., self-harm, suicide attempts or impulsive behavior) also to improvements in internal BPD-symptoms (e.g., chronic dysphoria, loneliness or feeling empty), as well as associated difficulties, including global psychosocial functioning, comorbid disorders, and quality of life. Thus, the outcomes of this study address not only BPD-symptoms but also the quality of life, psychosocial function and participation, general psychopathology and other psychiatric disorders. Further secondary hypotheses involve different effects of the two treatments in the different BPD symptoms and other secondary outcomes. Also, there might be different effects in subgroups of patients with BPD (e.g., different effects based on comorbid disorders or patterns of BPD symptoms) which will help to advance our knowledge about which treatment should be offered to which patient. More support from literature and discussion of the secondary hypotheses can be found in the discussion section.

We study moderating and mediating variables (e.g., sex, age, the severity of BPD-symptoms, comorbidity, traumatic experiences during childhood, experiential avoidance) through exploratory data analysis. Furthermore, we conduct a qualitative study on patients’ experiences with the two treatment methods.

The PRO*BPD study is conducted in the routine clinical setting of an outpatient clinic and responds to the call for ‘real world’-studies. As such, we use minimal exclusion criteria, set low barriers to patient participation and guarantee the clinical equipoise of the two treatment programs. Thus, we include BPD patients with the entire range of mental disorder comorbidity and high BPD severity and minimize resistance for participating in a clinical trial with randomization.

## Method/design

### Design

The study design is a randomized trial with two active groups (a treatment program for 1.5 years with either DBT or ST). This trial includes an evaluation of clinical effectiveness, an economic evaluation and qualitative research on patients’ perspectives of the two methods. Independent assessors collect data to evaluate the clinical effectiveness and cost-effectiveness with semi-structured interviews and self-report measures on BPD-specific symptoms, general psychopathology and BPD-associated psychopathology (e.g., dissociation, emotional dysregulation, depression), the course of comorbid disorders, quality of life, psychosocial functioning and participation and administer a structured interview to assess costs. Assessors are blind to the patients’ treatment condition for all outcome measures, only for assessment of treatment costs blinding is not possible. Data are collected at baseline and every six months until the end of treatment and continues 6, 12 and 24 months after the end of therapy as a naturalistic follow-up. In this follow-up phase, patients will be provided with supportive care in our center as indicated and can seek additional treatments outside of our center if they believe that they need more help. The data will include treatment participation in the follow-up-phase. Complete allocation concealment is not possible in a psychotherapy effectiveness study. To reduce bias, we employ partial allocation concealment by shifting the patients’ focus from the comparison of the two treatment methods. Because there is a comparison, patients could believe that one of the two methods is better than the other, which could lead to bias. Thus, the patients are not aware of the primary hypothesis in this study. The patients are informed (in the written and verbal patient study information) that the primary intention of the research is on the processes and predictors of therapy outcomes and that randomization is necessary for an unbiased distribution of participants across the two treatments to allow for unbiased tests of predictors and processes.

**Fig. 1:**
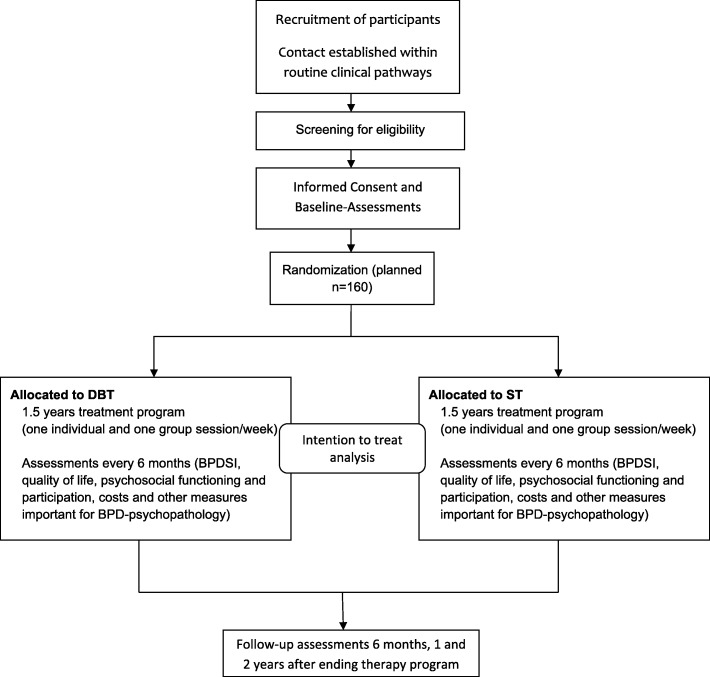
Flow chart of the study design. DBT = dialectical behavior therapy; ST = schema therapy, BPDSI = Borderline Personality Disorder Severity Index; BPD = Borderline Personality Disorder

The PRO*BPD trial adheres to the SPIRIT guidelines and methodology [56]. For an overview of the study design, see the flow chart for enrollment, intervention, and assessment that is shown in Fig. 1.

#### Recruitment and setting

The PRO*BPD study is performed in the outpatient clinic of the Department of Psychiatry and Psychotherapy at Lübeck University, Germany. The Department of Psychiatry treats more than 450 BPD patients/year (internal statistics) and has extensive experience in treating and researching BPD [48, 55, 57–59]. DBT has been used in Lübeck since 1999, while ST has been offered since 2009. Patients are included via the standard pathways of care in the clinic (inpatient and outpatient clinics, emergency room, and patients sent from other departments in the University Hospital). Potential participants are verbally informed about the study and are provided with written information. If they agree to participate they complete a signed written informed consent and the patient is invited for a screening to assess the in- and exclusion criteria.

#### Participants

Patients aged between 18 and 65 years are eligible if they (1) have a primary diagnosis of BPD (diagnosed with the Structural Clinical Interview for DSM-IV for Axis II; SCID II-Interview), (2) have a BPD severity score that is greater than > 20 points on the Borderline Personality Disorder Severity Index (BPDSI), Version 4 [60], (3) are willing to participate in the study (informed consent), and (4) are willing and able to remain in the study for 1.5 years and reliably participate in therapy (no relocation plans etc.). Exclusion criteria are (1) a major psychotic disorder (lifetime diagnosis), (2) intellectual deficits (IQ < 85), (3) poor German language skills, and (4) acute severe substance dependence that causes severe cognitive restrictions during therapy and necessitates detoxification treatment. Participation is possible after detoxification and a four-week period of abstinence. Patients who have cannabis dependence can participate if they commit to working on abstinence during treatment. We decided on a limited number of exclusion criteria to ensure high external validity and the generalizability of the results.

#### Sample size

The primary variable for calculating the sample size is the BPDSI total score. The sample size calculation is based on the number of participants that are needed to detect a clinically significant difference between the two conditions. Although the meta-analyses on DBT demonstrated global changes in effect sizes (ES) of d = 0.5 [27] and ST had a higher change in ES for borderline-specific symptoms (d = 2,96 [45] and d = 2.81 [44]), these cannot reliably be compared because they reflect different samples and outcome instruments, and there has been no direct comparison between the two methods. Therefore, we chose a medium ES of d = 0.5 for detecting group differences for the sample size calculation. Detecting a difference with this ES could strongly influence clinical decision making. As such, *n* = 64 patients per condition are required to detect group differences with a Type I error of 5% and a power of 80% (Type II error = 20%). Assuming an attrition rate of 20%, we will recruit *n* = 160 patients.

#### Randomization

Patients are informed about both treatment approaches and are willing to participate in a random assignment. Randomization is conducted in two patient pairs with a 50–50% assignment to one of the two therapeutic modalities using the program BiAS 11.02 [61]. Randomization is stratified by gender to control for the gender distribution in each treatment modality.

### Treatments

#### Common framework

The structural conditions of both treatment programs are as similar as possible to guarantee clinical equipoise: In both groups, patients receive a combined program of one group therapy session (120 min) and one individual therapy session (60 min) per week for a maximum of 18 months. Patients receive 4–10 weekly individual sessions before beginning the 18 months combined program to become accustomed to their individual therapist and the treatment model, as well as to be prepared for the group sessions. The exact number of individual sessions, which a patient receives, preceding the combined treatment program, is determined by the availability of treatment slots of the individual therapist. With absences that are due to illness, holidays or other disruptions, we estimate that each patient has an average therapy dose of 65 individual sessions and 60 group sessions. The groups consist of a maximum of ten patients and are conducted by two therapists. Groups are offered in a semi-open format. Thus, when treatment slots for patients are available (e.g., because of treatment drop-out or early success of a patient), new patients will enter the group. Thus, this format compensates for dropout and allows new study patients to begin more quickly than in a closed group. The time spots where new patients enter the group will be planned between research and therapist team and are dependent on a variety of points: available treatment slots in the group treatment and a time point that is suitable from a therapeutic stance to include new patients (e.g. after ‘group holiday’ or termination of a specific therapeutic theme), availability of an individual therapist to offer weekly individual sessions with at least four sessions preceding entry of the patient in the group treatment and completeness of baseline assessments. Eligible patients who are not able to attend group sessions due to family, professional or educational duties are included in the trial and are provided individual therapy only. In both conditions, patients can call their individual therapists outside of the sessions for support (a major component in DBT and to a lesser degree in ST). However, telephone support by the individual therapist is limited to office hours (which is normally not the case in DBT) to respect the participating therapists’ personal boundaries and to provide the same conditions for each patient. Outside of office hours, the psychiatric emergency service of our department is available for patients at any time. All patients also receive psychiatric management in our center. Additional medication is prescribed as needed according to national guidelines. According to our experience with BPD patients, this will reduce pre-existing polypharmacotherapy. Medication will be statistically controlled for in the data analyses.

#### Therapists, training, and supervision

Both experimental therapeutic approaches have a written protocol [22–24, 52]. Treatment is provided by a mixture of advanced DBT and ST therapists and therapists who are new to ST or DBT, but are experienced in CBT; with training before administering the treatment and learning the method under close supervision. Therapists have been and will be continuously trained by local and external certified specialists for the specific method in several workshops. We will assess the level of training and experience in the respective treatment method. For both therapies, there will be weekly supervision sessions under the direction of the locally approved supervisors (DBT: VS., ST: EF.) as well as team meetings. In the DBT condition, these team meetings follow the rules for DBT consultation teams (see below). For both methods, there are adherence scales to assess for therapy integrity [62, 63]. However, the psychometric evaluations of these adherence scales are not published yet. Video recordings are used for supervision and adherence ratings. Adherence will be rated in a random selection of 10% of session tapes from different treatment stages by trained raters who are blinded to the condition. Calibration checks of the raters on a subsample of these tapes will be conducted (at least 20 tapes, 10 DBT and 10 ST tapes, will be double-rated with both scales).

#### Treatment models and primary treatment strategies

*Dialectical behavior therapy (DBT)* was developed by Marsha Linehan in the late 1980 from a CBT background and views emotional dysregulation as the core of BPD. Problematic behaviors, such as self-injury, suicide attempts, substance abuse, binge eating, dissociation, or impulsivity are dysfunctional ways of regulating emotions. Therefore, the primary focus of treatment is to teach patients functional skills to accept and regulate their emotions. The dialectical perspective underlying the therapeutic process simultaneously involves accepting current difficulties and changing the problem behavior through skills acquisition. DBT uses a broad range of cognitive and behavioral treatment techniques to foster skills acquisition.

In group sessions (‘skills training groups’), patients learn the four DBT modules (mindfulness, distress tolerance, emotion regulation, interpersonal effectiveness), which all seek to improve emotion regulation skills, and are encouraged to train these skills regularly.

The individual therapist supports the patient in implementing the skills that were learned in the group, assists with troubleshooting and removes obstacles to change. Problem behavior is worked through based on a target hierarchy that is organized according to the threat posed to the patient from the behavior and the general goals of DBT therapy. The highest priority is acute life-threatening behaviors and serious self-injuries. The second priority is for behaviors that impede the therapy, while the third priority area addresses behaviors that impair quality of life. Important change strategies include, for example, analyzing problem behavior using behavioral analysis, or teaching behavioral skills for emotion and stress regulation and interpersonal relationships. To improve self-regulation and awareness, patients maintain stress diaries, emotional logs and weekly diaries (“Diary Cards”). With contingency management, adaptive behaviors are reinforced while modifying problem behaviors.

DBT telephone coaching is provided in crisis situations. As such, the patient can call his individual therapist during a crisis and receive support in applying suitable skills. The therapists meet weekly to support each other in providing DBT in a DBT consultation team [22, 23, 55].

*Schema therapy (ST)*, developed by Jeffrey Young, like DBT has its roots in CBT, but also draws ideas and techniques of other theoretical orientations (e.g., attachment theory, psychodynamic and experiential therapies such as Gestalt therapy). ST assumes that aversive childhood experiences and the frustration of basic childhood needs (e.g., secure attachment, love, attention or autonomy) lead to the development of dysfunctional maladaptive schemas (basic mental representations of the self, relationships to others, and the world). To address the related emotional distress, early coping strategies (surrender, avoidance, overcompensation) are developed to block access to feelings and needs and hinder the development of healthy interpersonal skills and closeness. Subsequently, these needs cannot be met later in adult life.

BPD relies almost exclusively on the disorder-specific mode model [64]. A mode is a specific state that influences emotions, cognitions, bodily reactions and behaviors in a given situation. The following modes are characteristic of BPD: (A) *abandoned/abused child mode*, which is associated with strong emotions, such as sadness, loneliness and fears of abandonment; (B) the *angry, impulsive child mode* is reflected in angry outbursts, hostility or impulsive behaviors; (C) the *punitive parent mode* is characterized by self-hatred, shame, self-devaluation, and self-punishment; (D) the *detached protector mode*, in which BPD patients attempt to detach from emotional pain by maintaining distance from other people and avoiding or distracting from emotions with e.g., self-harm, dissociation, substance abuse, binge eating or social withdrawal; (E) the healthy modes (*healthy adult mode and happy child mode)*, which are often weak at the beginning of treatment. Additional maladaptive modes can be added to the participant’s mode case conceptualization as indicated (e.g., a patient with narcissistic traits might have an additional overcompensating coping mode in his/her case conceptualization), which will also be addressed in treatment.

The primary therapeutic goal of ST is to help patients to have their needs met in their current life and to change maladaptive schemas. To achieve this goal, specific tasks are pursued for every mode: care for, soothe and comfort the vulnerable child modes to meet frustrated needs; help the angry child modes deal with anger, combat the punitive parent and reassure the detached protector mode, so that the patients can reduce their avoidance strategies and learn healthier strategies for managing emotions and relationships. Ultimately, the most important goal is to strengthen the healthy modes during the entire treatment process.

To achieve these goals, there are cognitive, experiential, and behavior-oriented interventions, and the working alliance is specifically designed as ‘limited reparenting.’ This suggests that the therapist –within the boundaries of a professional therapeutic relationship- behaves as a ‘good parent’ towards the patient to create an antidote for aversive interpersonal childhood experiences. Similarly, the ST group is designed as a ‘group family’ with the patients as the ‘siblings’ and the two therapists as the ‘parents’ [24, 25, 55].

#### Similarities and differences between DBT and ST

Both treatments share a cognitive-behavioral background, however; the approach is very different in practice. The primary differences are: In DBT, skills for stress and emotion regulation, mindfulness and interpersonal skills are taught using cognitive and behavior-oriented therapeutic techniques and the primary treatment focus is on the present. Patients are encouraged to practice the new strategies on a regular basis. In contrast, ST has a strong emphasis on early development and experiential techniques, such as imagery rescripting and chair dialogues, which are extensively used to change the patient’s emotional experiences. The DBT therapist acts as a ‘coach’ for the patient, while the ST therapist acts, to a limited extent, as a ‘good parent’ and uses interpersonal problem patterns that arise within the therapeutic relationship to change modes. For a detailed description of the similarities and differences between DBT and ST see [55]. Table 1 summarizes the main differences between the two methods.

**Table 1:** Main differences between Dialectical Behavior Therapy (DBT) and Schema therapy (ST)

	DBT	ST
Case conceptualization	Focus on connection between emotion regulation skills deficit and dysfunctional behavior; emotion dysregulation is the central problem	Case conceptualization uses the mode concept; the frustration of basic needs and trauma in childhood leads to the development of maladaptive schemas and modes. For each individual, there is a case conceptualization in schema mode terms that fits the patient’s profile.
Focus on childhood experiences	Primary focus on the present, focus on childhood experiences mainly in the context of validating emotional difficulties (level 4 validation)	Full integration: Current problems are associated with childhood experiences; psychoeducation for basic needs, the development of schemas and modes, emotionally processing aversive childhood memories to change the meaning of early experiences that underlie the schemas
Trained skills	Emotion regulation, stress tolerance, mindfulness, interpersonal skills	Awareness of one’s own needs, schemas and modes. Although the functional expression of emotions, needs and assertiveness is encouraged, there is no explicit skill training in these areas.
General therapeutic strategies	Validation strategies Commitment strategies Dialectical strategies (balance between acceptance and change, pro-contra lists) Major use of cognitive and behavioral techniques	Special focus on experiential (esp. imagery rescripting and chair-dialogues) and relational techniques (limited reparenting, empathetic confrontation) Mode-specific use of cognitive and behavioral techniques
Analysis of problem behaviors	Chain analysis based on the DBT model for each type of problem behavior; hierarchy of problem behaviors; focus on obvious and threatening problem behaviors, such as suicide attempts, self-harm and impulsive behavior	Mode analysis/chair dialogues or imagery work to understand problematic situations from the perspective of the mode model, focus on obvious problem behavior, as well as “hidden” problem behaviors, such as avoidance or surrender
Structure of the individual therapy session	Fixed structure with a “crisp beginning” that includes a diary card, processing topics according to the DBT goal hierarchy, and focusing on emotions	No fixed structure specification, flexible hierarchy depending on the dominating mode and the frustrated needs
Structure of the group session	Homework and goal-related opening and closing rounds, teaching skills from the DBT modules with a fixed manual; focus on cognitive and behavioral therapeutic techniques	Begins with safety imagery, topics depend on the dominating mode; designed as a “group family” to create corrective experiences; a primary focus on experiential and relational techniques
Addressing self-injury	Fixed procedures according to protocol based strategies, top priority in goal hierarchy; self-injuries are usually discussed with behavioral analysis before addressing other issues	No fixed structure specification, and does not need to be treated as a first priority (only if highly threatening); therapeutic intervention is directed at the mode that underlies the self-injury
Addressing interpersonal problems of the patient	Psychoeducation for interpersonal skills; behavioral training with standard and individual role-play exercises	Understanding interpersonal problems by using the mode model; interpersonal problem patterns normally also arise in the therapeutic relationship and are directly addressed, focus on expressing ones’ own needs and emotions; role-plays as needed but often in a later stage of therapy
Addressing emotional problems	Comprehensive psychoeducation in the modules for emotional management; mindfulness and acceptance of emotions; decisions about whether one should act according to or opposite from the emotion; emotion processing with the help of emotion protocols (cognitive approach)	Promotes experiencing emotions in a safe way; focus on needs (e.g., “What do I need when I’m sad?”); validating emotions and needs, when possible, fulfilling the need through limited reparenting, the empathetic confrontation of experiential avoidance that is displayed in the coping modes, experiential interventions, primarily imagery rescripting and chair dialogues, aim to develop corrective experiences
Developing the working alliance	Therapist acts as a ‘coach’ for the patient; the therapeutic team is at eye level with patient	Therapist acts, to a limited extent, as a ‘good parent’ with ‘limited reparenting’; uses the working alliance to change modes
Mindfulness training	Central role; non-judgmental attitude is promoted	Not included in ST
Skills training in distress tolerance	High priority; psychoeducation, developing a skills chain for stress regulation	Limited use, primarily for emergency situations at the beginning of therapy

### Clinical effectiveness study

#### Assessment and outcome measures

Before randomization, the patients are assessed at baseline. Baseline assessments are conducted at four assessment points over a period of approximately three months with one assessment point (assessing BPD severity and costs) directly before randomization. However, the timing of the baseline assessments will be adjusted based on the availability of treatment vacancies, e.g., if a treatment slot is available, the baseline assessment can be moved to a minimum time of one month to include the patient. On the other side, there can be a waitlist for inclusion and the baseline assessments will be performed over a longer time, with a maximum of a one year wait for treatment initiation. In this case, at the start of the waiting period, a check of in- and exclusion criteria is done, baseline assessments are performed not longer than six months ahead of treatment start. A re-assessment of the primary outcome, the interview for BPD severity, is performed, if the first baseline assessment of these measures is longer than three months ahead of treatment start. Assessments include PC-based questionnaires and semi-structured interviews. At baseline, in addition to the measures that are described below, there is a demographic questionnaire and the Childhood Trauma Questionnaire (CTQ). The CTQ assesses self-report childhood maltreatment experiences across five dimensions: emotional, physical, and sexual abuse as well as emotional and physical neglect. The original version and the German translation have good psychometric properties [65, 66].

Data are assessed at baseline and 6 (M1), 12 (M2) and 18 months (M3) after treatment initiation and 6 (M4), 12 (M5) and 24 months (M6) after the end of therapy for a naturalistic follow-up. An overview of the instruments at each assessment point is provided in Table 2.

**Table 2:** Instruments at each assessment

	Baseline^b^	Assessment points during the treatment program			Assessment points during Follow-up		
		6 months	12 months	18 months	6 months	12 months	24 months
BPDSI-IV	**••**	**•**	**•**	**•**	**•**	**•**	**•**
SCID I	**•**			**•**		**•**	**•**
SCID II	**•**			**•**		**•**	**•**
CTQ	**•**						
Demographics	**•**			**•**		**•**	**•**
WHODAS 2.0	**••**	**•**	**•**	**•**	**•**	**•**	**•**
WSAS	**•**	**•**	**•**	**•**	**•**	**•**	**•**
EuroQol-5D	**•**	**•**	**•**	**•**	**•**	**•**	**•**
WHOQOL-	**•**	**•**	**•**	**•**	**•**	**•**	**•**
BPD	**•**	**•**	**•**	**•**	**•**	**•**	**•**
QTF	**•**	**•**	**•**	**•**	**•**	**•**	**•**
DSS	**•**	**•**	**•**	**•**	**•**	**•**	**•**
BSI	**•**	**•**	**•**	**•**	**•**	**•**	**•**
QIDS	**•**	**•**	**•**	**•**	**•**	**•**	**•**
DERS	**•**	**•**	**•**	**•**	**•**	**•**	**•**
RSQ	**•**	**•**	**•**	**•**	**•**	**•**	**•**
WAI	**•** ^a^	**•**	**•**	**•**	**•**	**•**	**•**
DBT-WCCL	**•**	**•**	**•**	**•**	**•**	**•**	**•**
YSQ	**•**	**•**	**•**	**•**	**•**	**•**	**•**
SMI	**•**	**•**	**•**	**•**	**•**	**•**	**•**
Cost interview	**•**	**•**	**•**	**•**	**•**	**•**	**•**

*Abbreviations*: *BPDSI-IV*=Borderline Personality Disorder Severity Index version IV, SCID I=Structured Clinical Interview for DSM-IV Axis-I Disorders, SCID-II=Structured Clinical Interview for DSM-IV Axis-II Disorders, *CTQ* = Childhood Trauma Questionnaire, *WHODAS 2.0* = World Health Organization Disability Assessment Schedule 2.0, *WSAS* = Work and Social Adjustment Scale, EuroQol-5D = European Quality of Life questionnaire-5 dimensions, *WHOQOL* = World Health Organization Quality of Life questionnaire, *BPD* = Borderline Personality Disorder checklist, *QTF* = Questionnaire of thoughts and feelings, *BSI*=Brief Symptom Inventory, *QIDS* = Quick Inventory of Depressive Symptomatology, *DERS* = Difficulties in Emotion Regulation Scale, *RSQ* = Rejection Sensitivity Questionnaire, *WAI* = Working Alliance Inventory, applied in an individual and a group version

^a^assessed after third session, *DBT-WCCL* = Dialectical Behavior Therapy Ways of Coping Checklist, *YSQ* = Young Schema Questionnaire, *SMI*=Schema Mode Inventory

^b^Baseline consists of five assessments

•Instrument is assessed at the respective assessment point, ••BPDSI, WHODAS 2.0 are assessed twice at baseline

Due to the long duration of the study a team of blind raters will be needed to cover all assessments. The rater team consists of clinical psychologists with a completed further education in psychotherapy or an advanced state of this education as well as master students of clinical psychology who receive standardized training for the primary outcome interview and the WHO global functioning interview (WHODAS). The training for students and new clinical psychologists includes 2 to 3 interview sessions as an observer and a minimum of 2 interview sessions under life-supervision. Raters are only allowed to start with self-dependent interviews if the differences in ratings are reduced to a maximum of a one point difference not exceeding three items, and the performance of the interview is correct. Regular supervision will be offered for the raters as well as re-analyses of audio recordings to keep up the inter-rater reliability. The comorbid diagnosis will be assessed with the SCID I and II by clinical psychologists with a completed further education in psychotherapy or are in an advanced state of this education. These raters are well experienced in the use of the SCID due to specific SCID training and to their clinical practice. Participants and their therapists are informed of the participants’ scores on all instruments except for the cost assessment and the working alliance inventory (so that patients also report negative feelings towards their therapists). This is standing practice (regularly assessing progress or lack of progress) and is used as feedback to help to improve treatment.

##### Primary outcome measure

The primary outcome is BPD severity, which is assessed with the total score of the *Borderline Personality Disorder Severity Index version IV (BPDSI-IV)*, a semi-structured interview that rates all facets of BPD pathology. It assesses frequency and severity for the 9 DSM-IV BPD symptoms over the prior three months. The total score ranges from 0 to 90. The scores on the BPDSI-IV subscales provide information on the severity of each of the nine dimensions of BPD. The BPDSI-IV has excellent psychometric features (Cronbach’s alpha = .85; interrater reliability .99, high validity and sensitivity to change) [67, 68]. A cutoff of 15 points has been empirically shown to differentiate people who have BPD from people who do not have BPD; our inclusion criterion of > 20 has been used in several studies [45, 47, 50], as it reliably distinguished BPD from non-BPD PDs, and indicates severe BPD that is in need of treatment.

##### Secondary outcome measure

Secondary outcomes are assessed through interviews and self-report:

Psychosocial Functioning and Participation is administered as an interview and is assessed with the *World Health Organization Disability Assessment Schedule 2.0 (WHODAS 2.0)* [69], a general measure of functioning and disability in major life domains, including understanding and communication, getting around, self-care, getting along with others, life activities and participation in society. In addition, the *Work and Social Adjustment Scale (WSAS)* [70] a self-report instrument that assesses functional impairment in the domains of work, household, social leisure, private leisure, family, and relationships is used. The WSAS is reliable, valid and change-sensitive with different patient samples [71, 72].

Quality of life is assessed with two, well-established self-report questionnaires: the World Health Organization Quality of Life Questionnaire (WHOQol) [73] and the EuroQol-5D (EQ-5D) [74]. The WHOQol is a valid and reliable measure that assesses the quality of life in the two weeks prior the assessment for several domains (physical health, psychological health, social relationships, environment, positive feelings, negative feelings, and self-esteem). The EQ-5D measures health-related quality of life on five dimensions (mobility, self-care, activity, pain/discomfort and anxiety/depression). For the cost-utility analysis (see below), the profiles from these five health-related dimensions are assigned a value based on the social tariffs of the EQ-5D, the EQ-5D UK value set [75], to generate utilities. A German value set will be used if it becomes available. Utilities reflect a population’s preference for a specific set of health outcomes. The utilities from different time points are used to compute quality-adjusted live years (QALY) by multiplying the change in utility between the assessments by the duration of the period between the assessments. In addition, the EuroQol thermometer assigns a score that is between 0 and 100 for participant’s current subjective health status.

#### Comorbidity with other psychiatric disorders

The German version of the Structural Clinical Interview for DSM-IV (SCID-I and II-Interview) [76, 77] records comorbid psychiatric diagnoses according to DSM-IV criteria and will be administered at baseline, end of treatment and at one and two-year follow-ups. Previous studies demonstrated adequate to good interrater reliability. Diagnostic interviews (BPDSI-IV and SCID) are based on the DSM-IV classification system [78] because the DSM-5 [1] was not available when the study was planned and diagnostic instruments for the DSM-5 were not available at the beginning of the study.

#### Other BPD measures

The BPD checklist is a self-report scale that assesses the subjective burden that is caused by BPD manifestations. Research has established that this measure is suitable for use as a treatment outcome [79]. The Questionnaire of Thoughts and Feelings (QTF) assesses feelings, strategic cognitions, and assumptions that are characteristic of BPD. The QTF has shown excellent internal consistency (Cronbach’s alpha 0.91), high one-week test-retest reliability (*r* = 0.81) and high sensitivity to change over time in a sample of BPD patients during crisis intervention and eight months later [80, 81].

#### Dissociation

Dissociation is measured with the Dissociation Tension Scale (DSS), which is a self-report instrument that evaluates psychological, and somatoform dissociative features as well as the aversive inner tension that occurred in the past seven days. The DSS has high internal consistency (Cronbach’s alpha = 0.92), good validity, and sensitivity to change [82].

#### General psychopathology

The Brief Symptom Inventory (BSI) is an inventory of general psychiatric symptoms. The BSI was developed as a short version of the 90-item Symptom-Check-List (SCL-90-R), and has good psychometric properties [83]. The severity of depressive symptoms is measured with the self-rated 16-item version of the Quick Inventory of Depressive Symptomatology (QIDS-SR16), which has highly acceptable psychometric properties and is a treatment sensitive measure [84].

Difficulties in emotion regulation are assessed with the Difficulties in Emotion Regulation Scale (DERS), which has high internal consistency, good test-retest reliability, and adequate construct and predictive validity [85].

#### Rejection Sensitivity

The expectation of social rejection in close relationships is measured with the German version of the Rejection Sensitivity Questionnaire (RSQ) [86]. This questionnaire asks patients about 20 hypothetical situations in which they might experience rejections for their request for advice, help or companionship. Each situation is associated with two questions about (a) the level of concern and anxiety in this situation and (b) their expectation of the reaction (rejection or acceptance). The German version of the RSQ has high internal consistency (Cronbach’s alpha = 0.94) and test-retest-reliability (Pearson correlation after 2 weeks r_tt_ = .90). Rejection sensitivity was highly correlated with borderline-specific cognitions [86].

#### Therapeutic alliance

The Working Alliance Inventory (WAI) is one of the most commonly used and extensively validated measures of therapeutic alliance [87]. Many studies have shown that the quality of the working alliance predicts therapeutic success [88]. This was also demonstrated in the first outcome study on ST. This study found that ST had higher ratings on the WAI for both patients and therapists compared to TFP. Negative ratings were predictive of drop-out, while positive ratings at the beginning of treatment predicted subsequent clinical improvements [89]. In the PRO*BPD-study, we record the WAI from patients after the third session and at every subsequent assessment point. A group version of the WAI assesses the working alliance between the patient and other group members as well as between patient and group therapist. It is administered after the third group session and at every subsequent assessment point.

#### Method-specific measures

The Dialectical Behavior Therapy Ways of Coping Checklist (DBT-WCCL) [90], the Schema-Mode-Inventory (SMI) [91] and the Young Schema Questionnaire (YSQ) [92] serve as method-specific measures. The DBT-WCCL is a psychometrically sound measure that assesses DBT skill use [90]. Skill use mediates the decrease in suicide attempts and depression as well as the increase in control of anger in BPD patients who are treated with DBT [93]. The SMI is a self-report instrument that assesses the extent to which each of the 16 modes is present at the time of the assessment. It has an acceptable internal and high consistency for its subscales (Cronbach’s alpha ranges from 0.79 to 0.96), adequate test-retest reliability and moderate construct validity [91, 94]. The YSQ measures the presence or absence of 16 core maladaptive schemas at the time of the assessment. It has an adequate internal consistency and good reliability [95].

#### Treatment retention

If a patient drops out of treatment, the therapist completes a questionnaire that assesses the reasons for dropout. If the patient agrees, he is invited for the additional assessments that were scheduled at the beginning of treatment. Patients can stop the treatment program before 1.5 years and are viewed as an ‘early success’ if their BPDSI score is less than 15 and the therapist team agrees that the patient should stop the program. Patients may also be ‘pushed out’ of the treatment program e.g. if they continue to miss treatment sessions after interventions to increase attendance failed, if they deal with drugs or endanger the safety of other participants or therapists in other ways. We decided not to define explicit rules for a patient to be ‘pushed out’ of both conditions. It should be noted, that this is a difference from the standard DBT protocol, which states that missing four consecutive weeks of group sessions or four consecutive individual sessions will lead to treatment termination [22]. In the PRO*BPD trial a ‘push out’ is always decided by the therapist team and the local supervisors and is consistent with the respective treatment manual besides the above-mentioned divergence. In general, DBT has stricter rules about attendance, doing homework, the structure and content of therapy sessions (e.g., commitment to work according to the DBT hierarchy) compared to ST.

#### Statistical analyses of the clinical effectiveness study

Data analyses will be performed based on the intention-to-treat principle (including all patients regardless of whether they drop out from treatment or not) and will use all available data. Data for the primary and most secondary outcome measures will be analyzed with multilevel analysis to compare DBT to ST (mixed regression models for repeated measures). Time will be modelled according to the best model fit, e.g., a linear, logarithmic, or a segmented development of scores over time. The treatment by time interaction will be the primary test for treatment differences. We will use a piece-wise regression model if preliminary inspection of the data indicate that the time development during treatment has a different slope than during the follow-up period. If the time-development is better represented by a continuous equation (e.g., time, or SQRT (time), or log (time)) than we will do sensitivity tests of the treatment x time interaction for the treatment period, in addition to the test for the whole assessment period. We will also examine the between group differences at specific time-points, e.g., at the end of treatment and the final follow-up. For categorical outcome variables, counts appropriate forms of mixed regression will be chosen, with attention to non-normal residuals (negative binomial, Poisson, and gamma, etc.). Mixed regression methods will also be used to identify predictors/moderators that are related to treatment outcomes. Survival analysis will be used to analyze treatment retention.

### Cost-effectiveness study

#### Assessing resource consumption and productivity loss

Costs are assessed from several societal perspectives, including direct medical, direct non-medical and indirect costs, through structured face-to-face interviews (“Cost Interview”). The Cost Interview was used in another German study [10, 30] that examined the cost of BPD. Prior to the current study, modifications were made to improve clearness/comprehensibility, and additional items were incorporated into the Cost Interview. Items that are related to direct resource consumption include psychiatric and general hospital days, days in a psychiatric day clinic and assisted living programs, drug intake, visits to emergency rooms, outpatient psychotherapists, psychiatrists, general practitioners, medical specialists, occupational therapists, physical therapists, community based counselors and crisis centers. Items that are related to non-medical resource consumption include informal care, which occurs when significant others take over domestic tasks without payment. Also, we assess incidents of deviant behavior (e.g., traffic offenses, bodily harm) and their associated consequences, such as police operations or monetary compensation for damages. Productivity loss is captured with questions on employment status, the source of income, gross income, the number of weeks of employment, unemployment and work disability since the last assessment. Employed patients are asked about their average weekly work hours and the number of days that they were absent from work.

Resource consumption and productivity loss is retrospectively assessed. The baseline assessment occurs at the time of inclusion in the study and reflects the previous 12 months. During treatment and the 6 and 12-months follow-up, the Cost-Interview is conducted every six months. Further, the cost-interview is performed at 24 month follow-up about the previous 12 months (see Table 2). Wagner et al. found that it was difficult to clearly distinguish between BPD-related costs and costs due to other mental disorders because some symptoms (e.g., binge eating) are both a diagnostic criterion of BPD and other mental illnesses [10, 30]. Hence, the current study distinguishes between the costs that are due to psychological disorders and somatic diseases. First, the Cost-Interview is sent to patients for self-evaluation and to prepare for the interview appointment. In the interview appointment, missing items are assessed and it is determined whether resource consumption and productivity loss were due to psychological disorders or medical diseases.

#### Cost calculation and efficiency analysis

Costs are expressed in Euros using the 2015 price level. To calculate direct costs, we multiply resources by their corresponding unit cost (i.e., the cost of a particular medical or non-medical treatment). In contrast to other countries, such as Great Britain, there is no obligatory unit cost list in Germany. In the current study, unit costs are calculated based on Bock et al.’s [96] recent proposals. Bock et al. provided unit costs from a societal perspective for resources, including inpatient and outpatient medical care. Additionally, the authors showed how to update those unit costs on a yearly basis using regularly published sources. Indirect Costs are calculated according to the Human Capital Approach. Compared to the Friction Cost method, the Human Capital Approach is more applicable to BPD patients [97] because a sizable proportion of BPD patients are work disabled and work disability is not counted in the Friction Cost Method. We count productivity loss due to absences from paid work and productivity losses due to work disability. For patients with a paid job, we multiply the days absent from work by individual labor costs. Work disability-related costs are calculated based on the assumption that patients without BPD would pursue a paid job. Therefore, we multiply the national average monthly labor costs by the number of months that the patients were work-disabled.

The economic analysis is also based on the intention-to-treat principle. To determine the efficiency of DBT and ST, we will perform cost-effectiveness and cost-utility analyses. We will calculate two incremental cost-effectiveness ratios (ICER; the difference in costs between the two interventions divided by their difference in effectiveness). In the context of the cost-effectiveness analysis, we will calculate the costs per 10 BPDSI improvement points. For the cost-utility analysis, the costs per quality adjusted live years (QALY) will be calculated based on the EQ-5D questionnaire.

### Qualitative study on patient’s perspectives

Patients’ perspectives and experiences with the two treatment methods and with specific techniques of the specific treatment such as distress tolerance skills and opposite action for DBT or chair dialogues and imagery rescripting for ST are elicited through qualitative interviews, which will be performed with patients at different time points of treatment (after 5–8 months, 9–12 months and after treatment completion). All patients, no matter if they are completers or drop-outs, will be invited for the interviews and as many patients will be interviewed as necessary until saturation occurs (when no more information is added, and there is replication). At least 12 patients will be interviewed for each condition as a study demonstrated that *N* = 12 is usually enough to reach saturation [98]. The inclusion of treatment completers and drop-out, as well as the inclusion of patients at different time points, aims to assess a range of experiences and to obtain maximum variation sampling. All patients open for the qualitative study will participate in semi-structured in-depth interviews. Several primary components include: helpful and unhelpful aspects of the two methods, processes that facilitated change, and experience with specific ST and DBT techniques. Patients’ interviews will be recorded and transcribed. Transcripts of the interviews with patients will be analyzed for content following a specific manual [99] and using specialized software (MAXQDA). The nature of the qualitative studies is purely explorative.

## Discussion

This article described the study design of the PRO*BPD trial, which is a randomized trial that compares the clinical and cost effectiveness of DBT and ST for treating BPD offered in a 1.5-year outpatient treatment program. To the best of our knowledge, this is the first study that compares ST and DBT. Because these two methods are the primary CBT treatments for BPD, this comparison is long overdue. The primary hypothesis is that the two psychotherapeutic methods significantly differ in reducing BPD-severity (two-sided hypothesis). A two-sided hypothesis was chosen, because DBT and ST have never been directly compared before. Further, trials investigating the clinical effectiveness of one of the two methods are not comparable, as they used different outcome variables (DBT trials mainly focusing on suicidality, self-injuring and impulsive behaviors, while ST trials focusing on all nine BPD-criteria). Also, different samples of patients and doses of treatment have been investigated (for DBT mainly one year outpatient treatment, while ST mainly has been offered more extended treatment periods). Given these differences there is considerable uncertainty about which treatment is superior in reduction of overall BPD-severity.

A major criticism of the PRO*BPD trial might be, that we compare two bona fide psychotherapies and in line with the so-called ‘Do-do-bird-verdict’ according to the meta-analysis of Wampold et al. [100], a probable result might be, that there is no difference in the primary outcome between the two conditions. However, there is meta-analytic empirical evidence that casts doubt about a ‘Do-do-bird-verdict’ on psychotherapies for (B)PD. For instance, Wampold’s group has published a meta-analysis indicating that specific evidence-based psychotherapies do better than treatment-as-usual, and that some specialized psychotherapies do better than others [101]. Similarly, a meta-analysis demonstrated that specialized psychotherapies for BPD (the so-called ‘big-four’) do significantly better than control treatments [102]. Thus, the position that all treatments are equal in effectivity (and dropout), is not unequivocally supported by the data. Moreover, it is the present authors’ view that the question to what degree DBT and ST are similar in effectiveness and dropout rates from treatment, is an empirical issue which cannot be solved by a priori beliefs.

Because both methods use different approaches and techniques, the PRO*BPD trial is an opportunity to advance knowledge on the psychotherapeutic treatment of BPD and generate new hypotheses about differential effects and predictors. The two methods may lead to different effects on the secondary outcomes. For example, DBT focusses on acute life-threatening behaviors and severe self-injuries as the highest priority, and previous studies have shown that DBT has significant effects on reducing these behaviors [27]. Thus, we hypothesize that DBT reduces suicidal and self-harming behaviors better and faster than ST. DBT teaches patients functional skills to accept and regulate their emotions. Thus, we hypothesize that DBT will lead to better improvements of difficulties in emotion regulation (measured with the DERS). In contrast, ST is a transdiagnostic and more general approach that could lead to a better reduction in psychiatric comorbidity and more improvements in general quality of life.

The two methods may also perform differently for subgroups of BPD patients (e.g., different effects based on comorbid disorders or patterns of childhood abuse). For example, we hypothesize that patients who have high levels of self-injury and suicidality, as well as high impulsivity, will profit better from DBT, while we expect that patients who have comorbid avoidant PD and display more ‘hidden’ problem behaviors, such as avoidance, will benefit more from ST. Patients who have high scores of childhood traumatization (measured with the CTQ) and/ or comorbid posttraumatic stress disorder, will benefit more from ST because it directly addresses trauma. If we observe different effects of the two treatments for subgroups, it will assist in generating hypotheses on potential predictors of ST and DBT treatment success (i.e., ‘what treatment works better for whom?).

The PRO*BPD study has several strengths. First, a randomized trial that compares DBT and ST is unique to the field. Both treatments have a written manual [22–24, 52] and have previously been implemented in several scientific research trials. A common framework guarantees the clinical equipoise of the two methods. Both methods are administered by trained therapists under close supervision, and both therapist groups have a high allegiance to the specific method. Furthermore, for DBT and ST, recognized experts, trainers and supervisors (for DBT: VS, US, and for ST: EF, AA) are involved in planning and executing the study. Psychometrically sound and well-known outcome measures reflect important areas for treating BPD beyond pure symptom reduction (e.g., cost-effectiveness, psychosocial functioning and participation, comorbid disorders, and quality of life). Except for the Cost Interview, all outcomes, including the semi-structured interviews, are assessed by blind, independent and trained raters. The repeated measurements allow for close monitoring of change over time and the follow-up two years after treatment enables an examination of the long-term effects of the specific methods.

Most psychotherapy trials on BPD exclude patients who have comorbid diagnoses, such as severe eating, substance abuse, and antisocial or narcissistic personality disorders, despite high rates of comorbidity with BPD. A primary goal of the PRO*BPD study is to compare the effectiveness of the two treatments in routine clinical practice (‘real world study’) and address the real problems that are encountered in the outpatient care of patients who have BPD. The key points are (1) applying minimal exclusion criteria, (2) setting low barriers to participation for patients, and (3) waiving a comparison group with no treatment or treatment as usual in the community, so that severely ill patients can be included while minimizing resistance to randomization. These strategies guarantee representativeness of the study group, high ecological validity, and the practicability of patient recruitment. Furthermore, there is evidence for the primary efficacy of each of the two therapies, although there is no comparison data from realistic clinical studies. The ‘real world character’ is also promoted by implementing the two treatment programs in an outpatient clinic that provides general psychiatric health care.

Another strength of this trial is the multidimensional assessment of outcomes with a clinical and a cost-effectiveness trial as well as a qualitative interview study that examines the perspectives and experiences of patients who are treated with the two methods.

It is important to discuss several limitations and potential pitfalls. First, the sample size was calculated to reliably detect differential treatment effects for the primary outcome with a medium effect size or larger. However, with two bona fide psychotherapies, there could be smaller effects that might not be detected. However, the study contributes to building a database of treatments comparisons for BPD that meta-analytic techniques will use to document comparisons of different treatments. Second, we decided to provide two active groups and no control condition without treatment or treatment as usual. The study is deliberately designed as an effectiveness and not an efficacy study. However, when both treatments lead to improvements but do not have significantly different effects, the absence of an inactive control condition does not exclude the possibility that unspecific factors, such as time, may have led to the improvements. However, in addition to several serious ethical consequences, a control group would endanger the representativeness of the study group, which was explained above.

The PRO*BPD study is only conducted in one center at Lübeck University, which is a strength of the study because organizational/logistic issues and adherence to the research protocol is easier than in a multicenter trial. On the other hand, the monocentric design might have implications for generalizability and external validity. One possible barrier to the generalizability of our study population may be its unusually high severity and comorbidity compared to the general BPD population in Germany. This is because the University Hospital setting is utilized by a high number of patients who have complex BPD and high comorbidities, who cannot find access to therapy elsewhere (i.e., in private psychotherapy practices). Nevertheless, this specific group continues to lack access to sustainable treatment and causes immense health care costs due to their frequent and lengthy inpatient treatments and low psychosocial functioning.

Another point to consider is that the therapist teams have high enthusiasm and allegiance to the two methods and receive intensive supervision and training. On the one hand, this guarantees that the two methods are provided in the way they are supposed to be (‘adherence’). In contrast, one could argue that the time factor of training and weekly supervision is difficult to embed in general clinical practice, which may endanger the ‘real world’ character of the study. However, training and supervision are essential parts of both treatment programs and if this is necessary for successful treatment, training and supervision should be implemented everywhere in general clinical practice. Cost-effectiveness analyses will demonstrate whether this ‘extra time’ is cost-effective.

Another crucial issue is the treatment dose, as DBT and ST are applied with very heterogeneous doses and durations. In most DBT studies, the treatment duration was one year with one individual (60 min) and one long group session (180 min) per week. However, some studies tested shorter treatments, e.g., six months. In contrast, ST studies that examined individual ST for BPD had a treatment duration of 1.5 years or more with up to two weekly individual sessions. The one trial that tested group ST treatment offered only eight months of treatment with one weekly group session, but patients continued with individual TAU. We decided to provide the same treatment doses and durations for both conditions to better control for non-specific factors. For this trial, we identified a format that is feasible in the German health care system and is not too short to increase benefits for severely impaired patients. From our experience, one year of therapy is usually not adequate for these patients. Thus, we decided for a treatment duration of 18 months, with weekly individual and group sessions. This should suffice for applying the full range of ST and DBT treatment techniques without excessive redundancy.

Another limitation is that in some areas we had to deviate from the standard treatment manuals to guarantee clinical equipoise, to optimally deal with our severely ill patients or to adapt to the possibilities and limitations of the outpatient center of Lubeck University. There are some essential deviations from standard DBT that should be named: To protect the boundaries of the participating therapist we decided not to provide 24-h-telephone counselling, and we decided on another definition for treatment termination as explained in the methods section.

As another strength of the study treatment integrity will be checked However, the psychometric evaluations of the adherence scales are not published, which is a major limitation.

There are several other limitations due to the ‘real world’ character of the study. Because we rely on free treatment slots for individual and group treatments there will be variability in the range of 4–10 individual sessions patients will receive preceding the combined treatment program of group and individual and also the average of total individual and group sessions will vary. Some patients, who are not able to attend the group sessions, will also be part of the analyses. These patients will be equally randomized between ST and DBT-oriented treatment (DBT without group treatment is not standard-DBT). However, this is of course a limitation.

In conclusion, the PRO*BPD trial will significantly extend our knowledge on psychotherapy for BPD. Both DBT and ST hold promise for treating BPD. By examining the two methods’ clinical effectiveness over a broad spectrum of outcome parameters while performing an economic evaluation and assessing major stakeholder’s experiences, this study will significantly contribute to developing best practices for treating BPD and associated problems.

## Abbreviations

BPD: Borderline Personality Disorder
BPDSI-IV: Borderline Personality Disorder Severity Index version IV
BSI: Brief Symptom Inventory
CTQ: Childhood Trauma Questionnaire
DBT: Dialectical Behavior Therapy
DBT-WCCL: Dialectical Behavior Therapy Ways of Coping Checklist
DERS: Difficulties in Emotion Regulation Scale
EuroQol-5D: European Quality of Life questionnaire-5 dimensions
GST: Group Schema Therapy
ICER: Incremental cost-effectiveness ratios
QALY: Quality adjusted live year
QIDS: Quick Inventory of Depressive Symptomatology
RCT: Randomized Controlled Trial
SCID I: Structured Clinical Interview for DSM-IV Axis-I Disorders
SCID-II: Structured Clinical Interview for DSM-IV Axis-II Disorders
SMI: Schema Mode Inventory
ST: Schema Therapy
TFP: Transference Focused Therapy
WAI: Working Alliance Inventory
WHODAS 2.0: World Health Organization Disability Assessment Schedule 2.0
WHOQOL: World Health Organization Quality of Life questionnaire
WSAS: Work and Social Adjustment Scale
YSQ: Young Schema Questionnaire
